# Antioxidant Phytoconstituents From *Onosma bracteata* Wall. (Boraginaceae) Ameliorate the CCl_4_ Induced Hepatic Damage: *In Vivo* Study in Male Wistar Rats

**DOI:** 10.3389/fphar.2020.01301

**Published:** 2020-08-21

**Authors:** Ajay Kumar, Varinder Kaur, Kritika Pandit, Hardeep Singh Tuli, Katrin Sak, Subheet Kumar Jain, Satwinderjeet Kaur

**Affiliations:** ^1^Department of Botanical & Environmental Sciences, Guru Nanak Dev University, Amritsar, India; ^2^Indigenous Education and Research Centre, James Cook University, Townsville, QLD, Australia; ^3^Department of Biotechnology, Maharishi Markandeshwar (Deemed to be University), Ambala, India; ^4^NGO, Praeventio, Tartu, Estonia; ^5^Department of Pharmaceutical Sciences, Guru Nanak Dev University, Amritsar, India

**Keywords:** antimutagenicity, apoptosis, carcinogenesis, hepatoprotective effect, *Onosma bracteata*, phytoconstituents

## Abstract

*Onosma bracteata* Wall. (Boraginaceae) is a highly valuable medicinal herb that is used for the treatment of fever, bronchitis, asthma, rheumatism, stomach irritation, and other inflammatory disorders. The present study aims to explore the hepatoprotective potential of ethanolic extract (*Obeth*) from *O. bracteata* aerial parts against carbon tetrachloride (CCl_4_) which causes hepatic damage in the male Wistar rats. *Obeth* showed effective radical quenching activity with an EC_50_ of 115.14 and 199.33 µg/mL in superoxide radical scavenging and lipid peroxidation analyses respectively along with plasmid DNA protective potential in plasmid nicking assay. The *Obeth* modulated mutagenicity of 2 Aminofluorine (2AF) in the pre-incubation mode of investigation (EC_50_ 10.48 µg/0.1 mL/plate) in TA100 strain of *Salmonella typhimurium*. In *in vivo* studies, pretreatment of *Obeth* (50, 100, and 200 mg/kg) had the potential to normalize the biochemical markers aggravated by CCl_4_ (1mL/kg b.wt.) including liver antioxidative enzymes. Histopathological analysis also revealed the restoration of CCl_4_-induced liver histopathological alterations. Immunohistochemical studies showed that the treatment of *Obeth* downregulated the expression levels of p53 and cyclin D in hepatocytes. and downregulation in the Western blotting analysis revealed the downregulation of p-NF-kB, COX-2, and p53. HPLC data analysis showed the supremacy of major compounds namely, catechin, kaempferol, epicatechin, and Onosmin A in *Obeth*. The present investigation establishes the hepatoprotective and chemopreventive potential of *O. bracteata* against CCl_4_-induced hepatotoxicity *via* antioxidant defense system and modulation of the expression of proteins associated with the process of carcinogenesis in hepatic cells.

## Introduction

Global increase in the demand of industrial solvents in pharmaceuticals, paint and coating industry can raise the global solvent market to an estimated USD 12.31 billion by year 2026 as per Global Market Outlook report (2018). Recently, the use of industrial solvents has brought higher risks to both humans and animals. Industrial solvents cause environmental pollution and their acute and chronic exposure can cause cellular injuries and progressive genetic alterations (mutations) *via* activation of reactive oxygen species (ROS) with significant changes that may transform healthy cells to cancerous cells (Pizzino et al., 2017). The mutations caused alter the DNA which along with oxidative stress triggers destruction of tumor-suppressor genes and/or the stimulation of proto-oncogenes which contribute to the initiation of liver, breast, colon and prostate cancer (Maron and Ames, 1983; Canli et al., 2017).

The liver is the main organ essential for survival, which plays a crucial function in the body’s metabolism and detoxification process. The liver is a primary site in the body that is mostly involved in modulation and biotransformation of xenobiotic compounds mediated by cytochrome P_450_ (CYP) family of enzymes (Gu and Manautou, 2012). The chemicals like carbon tetrachloride (CCl_4_), acetylaminofluorene, thioacetamide, and polycyclic aromatic hydrocarbons are biotransformed into the transitional molecules and generate reactive oxygen species (ROS), including oxygen free radicals that induce hepatic damage (Karakus et al., 2011; Kaur et al., 2017; Kaur S. et al., 2019). Various hepatotoxic compounds and transitional radicals of oxidative reactions affect many proteins and encourage negative effects *via* oxidative stress (OS) or DNA damage (Rahal et al., 2014). The homeostatic balance of cellular redox is the main feature for combating the lethal insults of ROS, which are internally maintained by antioxidative defense systems. Any aberrant functioning to curb stress can lead to malfuctioning like cancer (Snezhkina et al., 2019). Carbon tetrachloride (CCl_4_) is a typical solvent which is used in the research for hepatic ailments. Firstly, liver can metabolize CCl_4_ by cytochrome P_450_ enzyme complex to produce toxic metabolites such as trichloromethyl free radicals (CCl_3_^•^) and trichloromethyl peroxyl radicals (CCl_3_OO^•^). These free radicals have capacity of eliminating hydrogen ions from fatty acids to induce lipid peroxidation. Hence, mutually CCl_3_^•^ and CCl_3_OO^•^ cause injury in the cell membrane, alteration in enzyme action and lastly cause hepatic damage or necrosis (Chao et al., 2019). The treatment of hepatic cancer still is one of the important tasks despite the newest developments in recognizing its biological basis. The deregulation of p53, COX-2, and NF-κB signaling is one of the central factors for the beginning of hepatic cancer (Alqahtani et al., 2019). p53 (a tumor suppressor gene) associated mutations are found in most of the hepatocellular carcinoma (HCC) (Aravalli et al., 2008). From the past few decades, the natural phytoconstituents from plants are recognized for restoring the health of humans (Che and Zhang, 2019). So, the diet rich in natural compounds has been shown to exert various healing effects *via* activating redox-signaling, phase-II detoxification enzymes, antioxidants, anti-inflammatory, and anti-carcinogenic properties (Wu et al., 2017).

*Onosma bracteata* Wall. family Boraginaceae widely knowns as ‘gaozaban’ is mainly distributed in India and Nepal in high altitude regions, spread in Jammu & Kashmir, Himachal Pradesh and Uttar Pradesh in north-western Himalayas (Ved et al., 2016). It is used as the main component for the preparation of drug in Unani and Ayurvedic medicinal system. It is usually used in Indian traditional medication due to its medicinal benefits *viz*. demulcent, diuretic, anti-aging, antioxidant, antibacterial, wound healing, antileprotic, spasmolytic, and tonic nature (Fareed et al., 2012). The entire parts of *O. bracteata* have pharmacological properties like anthelmintic, anti-inflammatory, antimicrobial, antiperoxidative, and radical scavenging (Albaqami et al., 2018; Imran et al., 2018; Farooq et al., 2019). It is reported that *Onosma* genus is a rich source of flavonoids, benzoquinones, naphthoquinones, shikonins, and onosmins (Kumar et al., 2013). However, to date, there are no records about its hepatoprotective activity.

Thus, we planned to explore the protective effects of *Obeth* from *O. bracteata* by using *in vitro* assays *viz*. antioxidant, antimutagenic and DNA protective potential. Further, *in vivo* studies were carried out to explore the hepatoprotective effects of *Obeth via* evaluation of serum marker enzymes, antioxidative enzymes and histopathological parameters. Modulatory effects on the expression level of p53, COX-2, NF-κB, and Cyclin D were studied using immunohistochemical (IHC) and Western blotting.

## Materials and Methods

### Chemical and Reagents

1-chloro-2, 4- dinitrobenzene (CDNB), 2- thiobarbituric acid (TBA), 5, 5-dithiobisnitrobenzoic acid (DTNB), Bovine Serum Albumin (BSA), Hydrogen peroxide (H_2_O_2_), malondialdehyde, nitro blue tetrazolium (NBT), and Silymarin were procured from Sigma (St. Louis, MO, USA). Carbon tetrachloride (CCl_4_), ethidium bromide (EtBr), low melting point agarose (LMPA), malondialdehyde (MDA), nicotinamide adenine dinucleotide (NADH), normal melting point agarose (NMPA), hydroxylamine hydrochloride, oxidized glutathione (GSSG), phenazine methosulfate (PMS), reduced glutathione (GSH), sodium pyruvate, and xylenol orange were obtained from HiMedia Pvt. Ltd., (Mumbai, India). The pBR322 (plasmid) DNA was purchased from Genei Pvt. Ltd. Bangalore, India. Cyclin-D (cyclin-dependent), p53 (tumor suppressor p53), COX-2, and Nf-κB antibodies were purchased from Cell signaling technology (Danvers, MA, USA). (PVDF) membrane (MDI, Ambala), ECL kit was procured from (Biorad, USA). Kits for analysis of serum parameters were purchased from Erba Mannheim (London, UK). The other chemicals used in the experiments were of Analytical grade.

### Collection, Identification, and Authentication of Plant Material

The aerial parts including stems and leaves of *Onosma bracteata* Wall. were collected from Herbal Health Research Consortium (HHRC) Pvt. Ltd. Amritsar, Punjab (India), a Government of India approved Drug analysis laboratory. Mr. Viney (Research Officer), at the HHRC Pvt, Ltd. identified and authenticated the plant material by analyzing organoleptic, microscopic and pharmacognostic characteristics. The specimen with accession no: GAZ-03 was submitted in the herbarium of HHRC Pvt. Ltd. Amritsar, Punjab (India).

### Extraction Procedure

The plant material was washed with running tap water and dried at 40°C. The material was crushed to powder form (2 Kg) and soaked with 80% ethanol by using the maceration method. The filtered material was evaporated using Rotavapor (Buchi Rotavapor R-210, Switzerland) and was dried to yield 120 g (6%) of the ethanolic extract (*Obeth*).

### Antioxidant Assays

#### Superoxide Radical Anion Scavenging Assay

The radical scavenging activity of *Obeth* was conducted according to the method suggested by Nishikimi et al. (1972), with little modifications. Initially, 0.06 M NBT and 0.156 M NADH were mixed to the different *Obeth* concentrations (25–400 μg/mL), along with the addition of 0.468 M PMS. In the control samples, *Obeth* was substituted by methanol in all of the above solutions. The mixtures were kept at room temperature for 20 min. In the incubation phase, the yellow-colored NBT was reduced to blue colour and the absorbance was recorded at 560 nm.

The percentage decrease of NBT was represented as:

$$\text{Percent change}=(({\text{OD}}_{C}-{\text{OD}}_{S})/{\text{OD}}_{C})\times 100$$

where OD_C_ = Absorbance of control solution.

OD_S_ = Absorbance of the sample solution.

#### Lipid Peroxidation (TBARS) Assay

For this experiment, the procedure was adopted as proposed by Halliwell and Gutteridge (1989), with a little amendment. The lipid origin (10% homogenous egg), 150 mM KCl, and *Obeth* (25–400 mg/mL) were combined in this assay. In place of *Obeth*, methanol was used as a control. To continue lipo-oxidation to the above reaction mixture, 10 mM FeCl_3_ was added followed by incubation at 37°C for 30 min. Subsequently, HCl solution (comprising TCA, TBA and BHT) was added to the mixture and the resultant mixture was heat treated for 1 h at 90°C accompanied by cooling and centrifugation at 2,500 rpm for 15 min. Lastly, the absorbance of pink coloured supernatant formed was recorded at 532 nm.

The estimation of the percent inhibition (anti-lipoperoxidation activity) was based on the formula below:

$$\text{Percent change}=(({\text{OD}}_{C}{\text{-OD}}_{S})/{\text{OD}}_{C})\times 100$$

where OD_C_ = Absorbance of control solution.

OD_S_ = Absorbance of the sample solution.

### Plasmid Nicking Assay

To determine the DNA protective ability of *Obeth* or capacity to scavenge the hydroxyl radical (^•^OH), the plasmid nicking assay was conducted as per the procedure given by Lee et al. (2002) with few amendments recommended by Robin et al. (2015). Different concentrations (25–100 µg/mL) of *Obeth* from *O. bracteata* were mixed with freshly prepared Fenton’s reagent (30 mM H_2_O_2_, 50 mM Ascorbic acid, and 80 mM FeCl_3_) and pBR322 plasmid DNA, followed by incubation for 30 min at 37 °C. The samples were resolved onto 1% (w/v) agarose gel using electrophoresis unit at 60 V for 1 h. For this assay, rutin (100 µg/mL) was used as a standard. Immediately, DNA bands were viewed with Gel Doc XR (Bio-Rad, USA), and the band density was determined by using LabImage Bioimaging Platform (Version: 4.1.1) software.

### Antimutagenic Activity

The antimutagenic potential of *Obeth* from *O. bracteata* was studied using Ames test, with two *Salmonella typhimurium* strains as per the method given by Maron and Ames (1983) with few amendments recommended by Kaur et al. (2000). The strains of *S. typhimurium* were procured from Microbial Type Culture Collection (MTCC), Chandigarh. Under sterile conditions, direct-acting mutagens, i.e., 4- Nitro-o-phenylenediamine and sodium azide were used for TA98 and TA100, respectively. The antimutagenic activity of *Obeth*, at different concentration levels (25–250 μg/0.1 mL/plate), was studied using two experimental approaches *viz*. co-incubation and pre-incubation modes to distinguish between the bio-antimutagenicity and desmutagenicity, respectively. Negative control contains only bacterial culture, *Obeth* and top agar which showed that *Obeth* did not exhibit mutagenic characteristics. In co-incubation mode, mutagen with *Obeth* was blended into 2mL of top agar containing bacterial culture whereas, in pre-incubation, the mixture of *Obeth* and mutagen was incubated at 37 °C for 30 min before adding to the top agar with bacterial culture. The positive control contains bacterial culture and mutagens which help to assess the dosage rate of toxicity/mutagenicity. *Obeth* antimutagenicity was also tested using S9-mix in the top agar as per the procedure suggested by Garner et al. (1972). The protocol includes a similar method as previously mentioned, except with the inclusion of S9-mix among other components of top agar for the activation of indirect acting mutagens. The reverting colonies were counted using a colony counter.

#### Antimutagenic Effect

Antimutagenic activity is defined by the percentage decrease in the number of colonies as shown below:

Percentage of Antimutagenic activity of Obeth=(a-b)/(a-c)×100

where,

a= number of mutagenic-induced colonies (positive control)

b= number of colonies induced with mutagen and different concentration of *Obeth*

c= number of colonies in (negative control).

### Hepatoprotective Activity

#### Animal Ethics Statement

This experiment was performed in compliance with the guideline of the Committee for the Purpose of Control and Supervision of Experiments on Animals (CPCSE), Ministry of Environment and Forestry, Government of India, and authorized by Institutional Animal Ethics Committee (IAEC), GNDU (approval number 226/CPCSEA/2014/06). As indicated by the IAEC, all rats were entitled to humane care.

#### Procurement and Care of Experimental Animals

Male Wistar rats, weighing between 150–240 g were purchased from the National Institute of Pharmaceutical Education and Research (NIPER), S.A.S. Nagar, Mohali, Punjab (India). The acclimatization of rats was conducted for one week in polypropylene cages containing husk in the aerated Central Animal House of Guru Nanak Dev University, Amritsar. Commercial rodent feed was provided for the rats, and water was given *ad libitum* with an environmentally regulated condition (55 ± 5 percent humidity, 23 ± 2° C) with a 12 h light/dark cycle photoperiod.

#### Experimental Design

After acclimatization, rats were divided into 7 groups (n=6). Group, I served as control; Group II intoxicated with 1 mL kg^-1^ CCl_4_ (mixed in same volume of olive oil *via* intraperitoneal (i.p.) doses, 3 days in a week) which induced liver mutilation and is mostly used for analyzing the hepatoprotective potential of various drugs (Ritesh et al., 2015; Zamzami et al., 2019); Group III was provided pretreatment of 50 mg kg^−1^ body weight (b.wt.) of silymarin (natural chemopreventive compound) for 21 days *via* oral route accompanied by CCl_4_ (i.p.) doses, 3 days in week without fasting of rats; Group IV was administered with an oral dose of only *Obeth* (100 mg kg^−1^ b.wt.) of *O. bracteata* as negative control; Group V was provided with the *Obeth* (50 mg kg^−1^ b.wt.) accompanied by CCl_4_ (i.p.) doses; Group VI was provided *Obeth* (100 mg kg^−1^ b.wt.) intoxicated with CCl_4_; Group VII was given *Obeth* (200 mg kg^− 1^ b.wt.) accompanied with CCl_4_ (i.p) treatment. The experimental concentrations of *Obeth* have been decided based on the literature report on *O. bracteata* in *in vivo* studies (Asif et al., 2019).

#### Blood Extraction and Serum Marker Enzyme Analysis

The rats were kept on fasting overnight before termination of experiment, the blood was extracted from retro-orbital venous puncture into heparinized tubes under moderate diethyl-ether anesthesia. The blood samples were centrifuged at 3,000 rpm for 20 min for the separation of serum. The extracted serum was used for examining the different hepatic markers enzymes (Serum Glutamic Pyruvic Transaminase (SGPT), Serum Glutamic-Oxaloacetic Transaminase (SGOT), Alkaline Phosphatase (ALP), total bilirubin, total protein, Albumin, urea, creatinine, and cholesterol) using Erba diagnostics kits with Bene Sphera autoanalyzer.

#### Animal Sacrifice and Preparation of Liver Homogenate

The liver was dissected after euthanizing by cervical dislocation. The collected liver was washed with ice-cold saline solution. After this, the liver was rinsed with the mixture of (150 mM KCl + 10 mM Tris-HCl) buffer. Finally, 10 percent (w/v) of dried liver homogenate was mixed with chilled Tris-KCl buffer and centrifugation at 2,500 rpm for 10 min. Supernatant was separated and used for further analysis.

### Biochemical Analysis of Liver Functions

#### Protein Quantification

The standard protocol given by Bradford, 1976 was used for the quantification of protein concentration in the liver. In this experiment, 900 µL of Bradford reagent was mixed with 100 µL of homogenate. The reaction solution was then kept for 30 min at room temperature and the absorbance was calculated on ELISA plate reader at 595/450 nm.

#### Estimation of Lipid Peroxidation

To check lipid peroxidation in liver homogenate, TBARS (thiobarbituric reactive species) procedure recommended by Devasagayam et al. (1983) was used. 2 mL of TBA solution was mixed to 500 μL of the sample. The mixture was fully stirred and incubated for 30 min at 80°C followed by centrifugation for 15 min at 2,000 rpm. Lastly, supernatant reading was taken at 532 nm and 600 nm. MDA was used as a standard for measuring the amount of TBARS represented as MDA value (MDA equal μmol/g of tissue).

#### Estimation of Lipid Hydroperoxides

Lipid hydroperoxide generation in liver homogenate was assessed as a method proposed by Jiang et al. (1992). 900 µL of Fox reagent (0.1 M Xylenol orange, 0.025M of H_2_SO_4_, 0.25 M of (NH_4_)_2_ Fe(SO_4_)_2_.6H_2_O, and 0.004 M of C_15_H_24_O) was mixed to 100 µL of liver homogenate. This reaction mix was kept for 40 min at 37°C and final reading was measured at 560 nm. The lipid hydroperoxide generation is shown as nM H_2_O_2_ equivalent/g of tissue.

#### Estimation of Reduced Glutathione Content (EC1.6.4.2)

The reduced glutathione content in liver homogenate was detected as per the protocol given by Anderson (1985) with little changes. Potassium phosphate buffer (200 µM, pH 8) and DTNB solution (600 µM) were mixed to 100 µL homogenate, preceded by incubation for 10 min and the absorbance was recorded at 412 nm. Glutathione was used as a reference for making a standard curve and total GSH content was expressed as µmol of GSH content/g of tissue.

#### Phase I Enzymes

##### Evaluation of Cytochrome P_450_ and P_420_ Content

Phase I enzymes were determined as per the method performed by Choi et al. (2003). In this protocol, the liver homogenate was added into two 96-well plates. In one plate 15 μL of sodium hydrosulphite (500 mM) was added in each well preceded with 30–35 bubbles of carbon monoxide. Another plate was saturated with only 30–35 bubbles of carbon monoxide without adding sodium hydrosulphite. The absorbance was observed at 420 nm, 450 nm, and 490 nm.

##### Cytochrome b5 Analysis

The cytochrome b5 (Cyt b5) quantities were analyzed as the method suggested by Omura and Sato (1964). In this experiment, 500 µL of liver homogenate was added to 4.3 mL of Tris-HCl buffer. After that, 50 μL of this mixture was poured onto two separate 96-well plates. Now, 50 μL of NADPH (500 mM) was added in each well of one plate and 50 μL of Tris-HCl in each well in the other plate followed by incubation for 20 min. The absorbance was measured at 409 nm and 424 nm. The enzyme activity was determined by using the extinction coefficient (6.22 mM^­1^ cm^­1^).

##### NADPH Cytochrome b5 Reductase

The NADPH Cyt b5 was determined using procedure recommended by Mihara and Sato (1972) with little modification. For this, 200 µL of NADPH (100 µM) and 200 µL of Potassium ferricyanide (200 µM) were mixed with 500 µL of potassium phosphate buffer (300 mM). The process was initiated with the addition of 100 µL of homogenate. The NADPH Phase II Enzymes

##### Glutathione-S-Transferase (EC 2.5.1.18)

Phase II metabolic isozyme glutathione-S-transferase (GST) was quantified as per the method suggested by Habig et al. (1974). In this experiment, 2.7 mL of sodium phosphate buffer (100 mM) was mixed with 100 µL each of 1-Chloro-2,4-dinitrobenzene (CDNB) (0.03 M) and 100 µL reduced glutathione (0.03 M) followed by incubation at 37°C for 5 min. After incubation, 100 µL of homogenate was added to initiate the reaction and enzyme activity was recorded at 340 nm at 30 s interval for 3 min.

### Antioxidative Enzymes

#### Catalase (CAT) (EC 1.11.1.6)

Catalase was analyzed in the liver homogenate as the method described by Aebi (1984) with little modifications. 300 µL of H_2_O_2_ (150 mM) and 2.6 mL of 50 mM potassium phosphate buffer (50 mM) were mixed thoroughly. After this, homogenate was added to initiate the reaction. Eventually, the absorbance was recorded for the catalase activity at an interval of 15 s, for 2 min at 240 nm.

#### Glutathione Reductase (GR) (EC 1.6.4.2)

A slight modification was done to assess the GR level in the samples using the protocol given by Carlberg and Mannervik (1975). In this assay, the reaction mixture was prepared by adding 1.2 mL of sodium phosphate buffer (200 mM), 200 μL oxidized glutathione (10 mM), 200 μL EDTA (20 mM), and 200 μL of homogenate. Finally, 200 μL NADPH (1000 µM) was mixed in a reaction solution and at an interval of 15 s, absorbance was measured at 340 nm for 3 min.

#### Lactate Dehydrogenase (EC 1.1.1.27)

To determine the lactate dehydrogenase (LDH) activity in liver homogenate, the protocol recommended by Kaur S. et al. (2019) with slight modifications was adopted. In this experiment, reaction mixture comprising of 2.3 mL of Tris­ HCl buffer (100 mM, pH 7.1), 300 μL of sodium pyruvate (100 mM), 100 μL Triton X (7.5%), and 300 μL of NADH (3 mM) was incubated at 30°C for 15 min. In this reaction mixer, 100 μL of homogenate was added and absorbance was measured at an interval of 10 s at 340 nm for 1 min.

#### Guaiacol Peroxidase (EC 1.11.1.7)

Guaiacol peroxidase activity in liver homogenate was determined as per the method suggested by Gee et al. (1970) with slight modifications. The reaction mixture was prepared by adding 75 μL (20 mM) of guaiacol solution to 25 μL 100 mM phosphate buffer with 100 µL of liver homogenate and then 25 μL of H_2_O_2_ was added. The absorbance was taken at 436 nm.

### Histopathological Assessment

To check the hepatocytes’ structural integrity, the isolated livers from the respective groups were fixed in a 10% buffered formalin solution, dipped in ethanol for dehydration and implanted in paraffin wax. The microtome was used to make sections of the livers (0.4 mm thick) using paraffin blocks and stained with H&E (hematoxylin and eosin) by the standard procedure. The slides were observed with a Nikon Eclipse E200 Compound microscope.

### Immunohistochemical (IHC) Examination

To evaluate the pattern of expression of p53 and cyclin D proteins (after treatment with CCl_4_ and *Obeth*) in hepatocytes, IHC analysis was performed. In this method, the liver sections (3-6 μm) were deparaffinized on glass slides. The sections were kept for 30 min in H_2_O_2_ (0.3% of methanol). The section slides were blocked with 5% skimmed milk for 30 min. Finally, slides were incubated overnight with anti-p53 and anti-cyclin D antibodies at 4°C. Slides were then washed twice with 1 x PBS followed by the addition of anti-rabbit IgG HRP- conjugated secondary antibody and incubated for 30 min and washed thrice with 1x PBS. The expression of proteins was developed by using 3-diaminobenzidine (DAB) for 2–3 min and washed with 1 x PBS. The slides were observed under a Nikon Eclipse E200, Japan microscope.

### Single-Cell Gel Electrophoresis (Comet Assay)

The comet assay (single-cell gel electrophoresis) was performed according to the protocol given by Collins and Dušinská (2002) with slight modifications. In this assay, 500 mg of rat liver was minced in trypsin solution and kept for 15–20 min at room temperature. The homogenate was passed through a muslin cloth to remove tissue debris followed by centrifugation at 2,500 rpm for 10 min to obtain a cell pellet. After that, 50 µL of cell suspension was added to a low melting point agar (LMPA) solution and transferred to normal melting point agar (NMPA) coated glass slides. The slides were kept in the fridge for 10–15 min. The slides were immersed in lysine buffer (100 mM EDTA, 10 mM Tris-HCl, 1% Triton X-100, 250 mM NaCl, 10 percent DMSO; pH 10) at 4°C for 3- 4 hr. Now slides were dipped in electrophoresis buffer for 10-15 min before starting electrophoresis (voltage = 25 V, current = 300 mA, time = 20 min). After electrophoresis, the slides were dried and kept in neutralization buffer for 15 min (thrice). After 24 h slides were stained with 100 μL (20 μg/mL) ethidium bromide and observed in the fluorescent microscope (Nikon, Tokyo, Japan). Per slide thirty cells were selected randomly to assess DNA impairment, tail length, tail DNA (%) and tail moment using Comet Assay Software Project (CASP) Lab version CASP1.2.3 beta 1.

### Western Blot Analysis

The liver was dissected and rapidly stored according to respective groups at -80°C. The samples were homogenized in RIPA lysis buffer followed by centrifugation at 12,000 rpm for 20 min and the upper transparent solution was then poured into new tubes. The quantity of proteins was analyzed using Bradford method. Equal concentration of protein (40 μg) was resolved by SDS-PAGE and transferred to PVDF membrane through western bloting. After that, the membrane was blocked using BSA (5% in TBST, 0.1% Tween-20) for 2 h at 25°C and kept overnight with antibodies against p53 (1:1,000), NFκB (1:2,000) and COX-2 (1:500). After that, the membrane was washed thrice with TBST and HRP-conjugated secondary antibody (1:1,500) was added followed by incubation for 2 hr at room temperature. ECL reagent was used to develop blots and imaging was done using Image-Quant LAS 4000, GE Healthcare. Band densities were quantified with Alphaease FC Software (version 4.0). β-actin (1:500) as endogenous control was used for normalizing the expression of the protein of interest.

### Phytochemical Analysis

#### HPLC Analysis

The filtered *Obeth* was analyzed for the presence of polyphenols using UHPLC Nexera system (Shimadzu, MA, USA), column (C18). The solvent used in mobile phase consisted of solvent A (0.2% acetic acid) and solvent B (methanol). The flow rate of 1.0 mL/min was sustained for 21 min of running time. The gradient flow used was: 20% B (0–10 min), 55% B (10–12 min), 70% B (12–14 min), 50% B (14–15 min), 40% B (15–17 min), and 30% B (17 –21 min). For testing, the injection volume of the sample was 10 μL with column temperature maintained at 25°C. The spectra were observed at 280 nm with a photodiode array (PDA) analyzer. The peaks of the compound were identified by comparing retention time and distribution of UV-VIS spectra of samples with those of standard compounds.

### Statistical Analysis

All results are represented as Mean ± SE of minimum three independent replicates. To compare difference among the means, one-way and two-way analysis of variance (ANOVA) with Tukey’s test was used at 5% confidence level (p-value ≤ 0.05).

## Results

### Antioxidant Potential of *Obeth*

**Figure 1:**
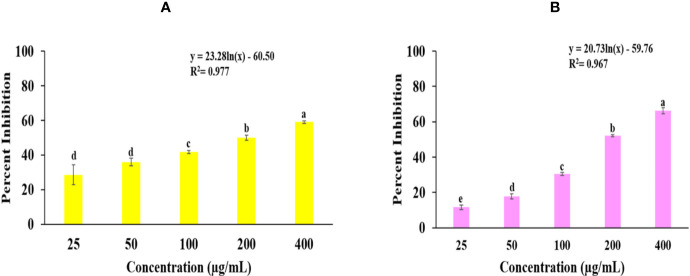
Graphs shows the *in vitro* antioxidant potential of *Obeth* from *O. bracteata*. **(A)** Superoxide radical scavenging assay. EC_50_ = 115.14 µg/mL; F-ratio = 187.19; HSD = 8.77. **(B)** Lipid peroxidation inhibition assay. EC_50_ = 199.33 µg/mL; F-ratio = 331.68; HSD = 5.89. Values are expressed as Mean ± SE at level of significance p ≤ 0.05. Data labels with different letters represent significant difference among them.

Superoxide radical scavenging and lipid peroxidation activity of *Obeth* is shown in **Figure 1**, **Table S1**. SRS assay there is dose-dependent increase in free radical scavenging activity *via* NBT inhibition with EC_50_ (115.14 μg/mL) whereas EC_50_ of Rutin was observed to be 46.18 µg/mL. The lipid peroxidation activity of Rutin and *Obeth* was EC_50_ (115.68 μg/mL) and EC_50_ (199.33 μg/mL) respectively (**Table S2**) exhibiting *Obeth* with better antioxidant activity as compared to standard rutin.

### Plasmid Nicking Assay

**Figure 2:**
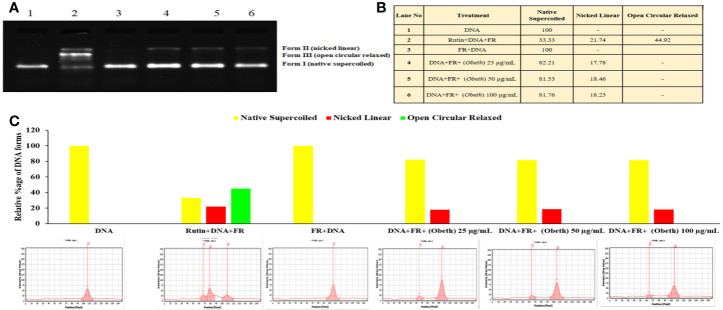
Effect of *Obeth* from *O. bracteata* on DNA protection against oxidative damage caused by Fenton’s reagent (FR) in DNA nicking assay. **(A)** Relative mobility of different forms of plasmid DNA exposed to *Obeth* extract in electrophoresis. Lane 1: Negative control (pBR322 DNA + distilled water); Lane 2: Positive control (pBR322 DNA + FR); Lane 3: pBR322 DNA + FR + 100 µg/mL rutin; Lanes 4, 5, 6: pBR322 DNA + FR + 25, 50, 100 µg/mL *Obeth* fraction respectively. **(B)** Densitometric analysis showing relative percentage of different DNA forms in plasmid after treatment with various concentrations of *Obeth* fraction in the form of table and **(C)** graph respectively (software used = Lab Image). CN = Control, FR=Fenton reagent.

DNA protective effect of *Obeth* at various concentrations *viz*. 25, 50, 100 µg/mL was demonstrated in **Figure 2**. It was observed that the *Obeth* exhibits quite strong radical scavenging properties with the highest tested concentration (100 μg/mL) on pBR322 plasmid DNA. The experiment exhibited the formation of nicked DNA because of hydroxyl (^•^OH) radicals produced by Fenton’s reagent. The various concentrations of *Obeth* showed a dose-dependent minimization of supercoiled DNA to open circular.

### Ames Assay

**Figure 3:**
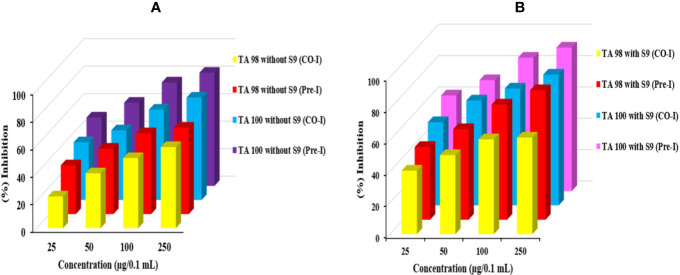
Graph represents the antimutagenic potential of *Obeth* using *S. typhimurium* in Ames assay. **(A)** Represents the TA100 and TA98 without S9 (Pre and Co-incubation). **(B)** Represents TA100 and TA98 with S9 (Pre and Co-incubation). **(A)** Represent the percent inhibition between co- and pre-incubation in TA98 and TA100 strain without S9. **(B)** Represent the percent inhibition between co- and pre-incubation in TA98 and TA100 strain with S9.

The antimutagenic potential of *Obeth* was tested against sodium azide and 2-aminofluorene mutagenicity in *S. typhimurium* strains as shown in **Table S3**. The *Obeth* in TA100 strain (-S9 mix) displayed a high inhibition rate (82.30% at 250 μg/0.1 mL/plate) showing strong modulation of genotoxicity of base-pair substitution mutagen sodium azide as compared to NPD (frame-shift mutagen) in TA98 tester strain **Table 1**. The *Obeth* exhibited high antimutagenicity potential for preincubation mode than in co-incubation approach without -S9 in both TA100 and TA98 as shown in **Figure 3A** and with +S9 mix in **Figure 3B**. Minimum EC_50_ values were depicted by the extract against 2AF mutagenicity in pre-incubation mode in TA100 strain as shown in **Table S3**. In case of TA98 strain also, *Obeth* showed good antimutagenic potential in pre-incubation experiments as compared to co-incubation approach (**Figure 3**, **Table 1**). The calculated EC_50_ value showed the high suppression of mutagenicity with a minimum EC_50_ value (29.51 µg/0.1 mL/plate) with TA98 (+S9) in the pre-incubation approach of treatment as shown in **Table S3**. The evaluation between all treatments, with and without S9, is shown in **Figure 3**, **Table S3** respectively. The assessment permits noticeable potential of *Obeth* +S9, even at minimum concentrations, as compared to -S9 treatments. Therefore, the complete investigation showed the conspicuous activity of *Obeth*, with S9 mix in pre-incubation approach. The findings were statistically evaluated by two-way ANOVA for statistical difference and the interactions as seen in **Table 1**, **S3**. The study revealed a statistically significant difference between co-incubation and pre-incubation methods of investigation, in addition to among different treatments used. So, the *Obeth* of *O. bracteata* exhibited marked antimutagenicity potential.

**Table 1:** Percentage inhibition of mutagenicity by *Obeth* extract of *O. bracteata* in TA98 and TA100 tester strains of *Salmonella typhimurium* with and without S9.

Treatment	Dose/0.1 mL/plate	TA 100 (without S9)% Inhibition	TA 98 (without S9) % Inhibition	TA 100 (with S9) % Inhibition	TA 98 (with S9) % Inhibition
**Co-incubation**	25 µg	42.01 ± 4.25^b^	23.07 ± 3.42^c^	53.02 ± 5.71^c^	40.40 ± 3.42^c^
	50 µg	50.69 ± 4.24^b^	39.92 ± 3.76^b^	66.85 ± 1.66^b^	50.35 ± 2.05^b^
	100 µg	65.71 ± 2.48^a^	50.98 ± 1.65^ab^	74.48 ± 2.59^ab^	60.37 ± 1.40^a^
	250 µg	74.47 ± 2.51^a^	58.97 ± 1.81^a^	83.37 ± 1.21^a^	61.51 ± 2.08^a^
**Pre-incubation**	25 µg	49.77 ± 2.88^c^	35.53 ± 2.79^c^	60.91 ± 2.01^c^	46.39 ± 2.45^c^
	50 µg	60.51 ± 2.06^b^	47.60 ± 2.76^b^	70.66 ± 3.44^b^	57.74 ± 1.79^b^
	100 µg	75.28 ± 2.91^a^	58.79 ± 1.74^a^	85.01 ± 1.11^a^	73.43 ± 3.45^a^
	250 µg	82.30 ± 1.63^a^	62.85 ± 3.33^a^	91.41 ± 1.26^a^	82.56 ± 2.24^a^

Values are represented as Mean ± SE, level of significance p ≤ 0.05. Means with different superscripts represent significant difference among them in all groups.

### Variations in Body Weight (b.wt.) of Animals

In the present study CCl_4_ treated group, the percentage fold decrease in b. wt. of rats was recorded after 3 weeks, and a statistically significant difference was observed in the b.wt. of CCl_4_ rats treated group (0.88-fold) relative to control group I (one-fold). Additionally, dose-dependent variability in the b.wt. of *Obeth* + CCl_4_ intoxicated groups was maintained b.wt. as the control group (**Table S4**).

### Serum Marker Enzymes

#### Effect of *Obeth* and CCl_4_ on SGOT, SGPT, and ALP Levels

**Figure 4:**
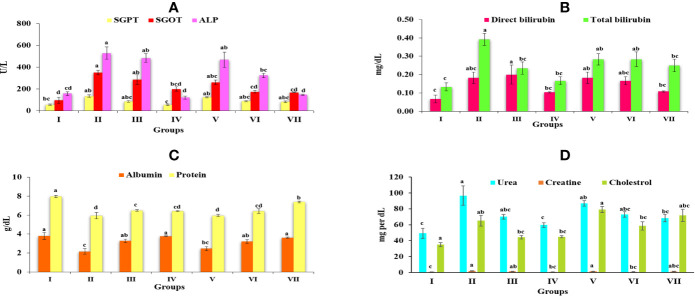
Graph represents the effect of *Obeth* of *O. bracteata* on level of serum enzymes level in CCl_4_-treated male Wistar rats. **(A)** Represent the levels of SGOT, SGPT, and ALP. **(B)** Represent the levels of direct bilirubin and total bilirubin. **(C)** Represent the levels of albumin and total protein. **(D)** Represent the levels of urea, creatine and cholesterol. Values are expressed as Mean ± SE at level of significance p ≤ 0.05. Data labels with different letters represent significant difference among them.

Group II (CCl_4_) significantly increased the amount of SGOT, SGPT, and ALP to 351.45 ± 21.01, 133.86 ± 29.88, and 529.16 ± 56.74 IU/L respectively in comparison with Group I (control) at 5% level of significance (p ≤ 0.05) (**Table S5**). Increased concentration of these enzymes in serum, highlighted the poor functioning of the liver and liver injury because of their seepage into the blood. However, the results showed that the treatment of *Obeth* minimized the amount of these enzymes relative to the CCl_4_ Group II. III and IV exhibited the amounts of SGOT, SGPT, and ALP in serum in the acceptable limits, signifying reduction in liver injury (**Figure 4A**).

#### Direct Bilirubin and Total Bilirubin Enzymes in *Obeth* and CCl_4_ Treated Rats

*Obeth* pretreatment followed by CCl_4_ doses in Groups V, VI, and VII was effective in maintaining natural level of direct bilirubin and total bilirubin disturbed in Group II (**Figure 4B**). In Group III and *Obeth* treated Group VII indicated an optimum capacity for regeneration by stopping a rise in these enzymes (**Table S6**).

#### Total Protein and Albumin Level in Serum

CCl_4_ stimulated changes in the hepatic enzymes resulting in decreased albumin (2.15 ± 0.3 g/dL) and protein (5.93 ± 0.34 g/dL) content that lead to hepatic injury (**Table S7**). Pretreatment of *Obeth* followed by CCl_4_ intoxication exhibited substantial protective capacity retaining the normal levels of these enzymes as identified in Groups V, VI, and VII (**Figure 4C**).

#### Effect of *Obeth* and CCl_4_ on Serum Proteins, Urea, Creatinine, and Cholesterol

The levels of urea, creatinine and cholesterol in the serum of various groups is given in **Table S8**. There was a significant increase in the level of urea, creatinine and cholesterol in CCl_4_ treated Group II. As depicted from the results of Group IV pretreated with *Obeth* followed by CCl_4_ showed a restored level of urea, creatinine and cholesterol, hence showing the recovered hepatic damage in a dose-dependent manner (**Figure 4D**).

### Biochemical Parameters

#### Estimation of Protein Content in Liver Homogenate

**Figure 5:**
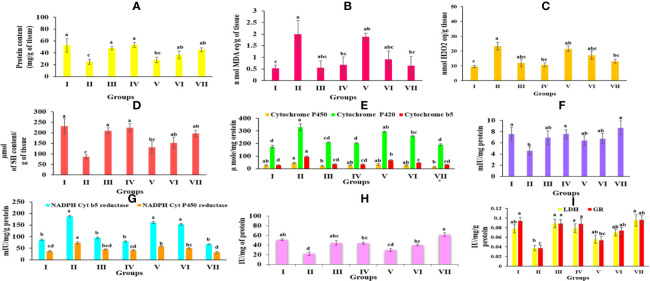
Graph represents the effect of *Obeth* of *O. bracteata* on level of enzymes in CCl_4_-treated male Wistar rats. **(A)** Represent the levels of protein in liver homogenates. **(B)** Represent the levels of malondialdehyde (MDA) content in liver homogenate. **(C)** Represent the levels of lipid hyperoxide in liver homogenates. **(D)** Represent the levels of reduced glutathione (GSH) in liver homogenate. **(E)** Represent the levels of NADH Cyt b5 and NADH Cyt P_450_ in liver homogenate of Wistar rats. **(F)** Represent the levels of GST in liver homogenate of Wistar rats. **(G)** Represent the levels of NADH Cyt b5 and NADH Cyt P_450_ in liver homogenate of Wistar rats. **(H)** Represent the levels of Catalase in liver homogenate. **(I)** Represent the levels of lactate dehydrogenase and glutathione reductase in liver homogenate. Values are expressed as Mean ± SE at level of significance p ≤ 0.05. Data labels with different letters represent significant difference among them.

The protein content in the CCl_4_-treated group (24.89 ± 4.16 mg/g tissue) was observed to decrease significantly as compared to the control group (52.74 ± 11.37 mg/g tissue) **Table S9**. Group IV, however, showed a high protein content of 53.38 ± 4.21 mg/g. On the other side, pretreated groups (V, VI, and VII) were shown to greatly recover the normal protein amount as seen in **Figure 5A**.

#### Estimation of Lipid Peroxidation in Liver Homogenate

The level of MDA content exhibited the level of lipid peroxidation. Group II revealed a significant increase (p ≤ 0.05) in the MDA content relative to the Group I. Group IV *Obeth* treated exhibited less concentration of MDA content (0.67 ± 0.33 nM MDA equivalent/g of tissue). However, pretreatment of *Obeth* followed by CCl_4_ resulted in a dose-dependent decrease in raised MDA content as seen in **Figure 5B** and **Table S9**.

#### Estimation of Lipid Hydroperoxide in Liver Homogenate

It was observed that the group treated with CCl_4_ displayed a substantial increase in the lipid hydroperoxide content (23.24 ± 2.54 nM equivalent H_2_O_2_/g of tissue) relative to the control group (9.43 ± 0.79 nM equivalent H_2_O_2_/g of tissue). Pretreatment by *Obeth* maximum dose showed to decrease CCl_4_ toxic impact by exhibiting a maximal decrease in lipid peroxide content **(****Figure 5C** and **Table S9**).

#### Reduced Glutathione (GSH) Content

The group administered with silymarin displayed substantial improvement in GSH content (209.75 ± 14.71 μmol of SH content/g of tissue) compared with CCl_4_-treated group with GSH content of 85.99 ± 9.57 μmol of SH content/g of tissue (**Table S9**). *Obeth* recovered the reduced amount of GSH effectively as close to that of the control group (**Figure 5D**).

#### Phase I Enzymes

As shown in **Table S10**, *Obeth* exhibited a dose-dependent effect on cytochrome P_450_, b5 and P_420_ levels in the liver homogenate. CCl_4_-treated group exhibited reduction in the level of cytochrome P_450_ (45.90 ± 5.07 μmol) and P_420_ (329.18 ± 26.63 μmol) as compared to the control group. Pre-treatment of *Obeth* followed by CCl_4_, showed increasing cytochrome levels P_450_ and P_420_ showing an important defensive effect against hepatic toxicity (**Figure 5E**). A rise in the production of cytochrome b5 (98.32 ± 5.74 μmol) in CCl_4_ treated group compared to the control group (29.34 ± 2.27 μmol) was observed. NADH cytochrome P_450_ reductase activity showed an increase in the CCl_4_-intoxicated group relative to the control group. Silymarin (45.27 ± 1.91 mIU) and *Obeth* Group IV (41.82 ± 3.23 mIU) showed same activity as compared to the control group of rats (37.23 ± 2.96 mIU) whereas group VII showed decrease in level of enzyme (32.47 ± 4.99 mIU) as shown in **Table S11**. Moreover, the CCl_4_-treated group exhibited an increase in the specific action of NADH cytochrome b5 reductase to 188.43 ± 3.86 mIU in comparison to the control group (86.40 ± 3.17 mIU) whereas group VII exhibited minimization in level of enzyme (67.20 ± 2.36 mIU) as shown in (**Figure 5G**, **Table S11**).

#### Phase II Enzymes

##### Glutathione S-Transferase (GST)

CCl_4_ treated group showed decreased GST behavior (4.59 ± 0.684 mIU/mg protein) compared to the control group (7.6 ± 1.14 mIU) (**Table S12**) (**Figure 5F**). However, it was observed that there was a significant increase in GST activity in group III, IV, and VII which showed enhancement of hepatic antioxidant enzymes to combat the increased level of reactive oxygen species.

### Antioxidative Enzymes

#### Catalase

As shown in **Table S13**, the catalase activity decreased in the CCl_4_-intoxicated group (21.92 ± 3.76 IU/mg protein) as compared to control group (50.76 ± 1.95 IU/mg protein). The higher pretreated dose of *Obeth* (200 mg/kg b.wt.) followed by CCl_4_ showed an increase in catalase function over the untreated group, thereby offering protective activity against CCl_4_ intoxication (**Figure 5H**).

#### Glutathione Reductase (GR)

The enzymatic expression of GR in the CCl_4_-administered group showed 0.03 IU of GR level as compared to control group (0.09 IU). The increased GR specific activity was detected in groups VI and VII which suggested *Obeth* has a protective role against hepatic damage done by CCl_4_ (**Table S13**, **Figure 5I**).

#### Lactate Dehydrogenase (LDH)

The level of lactate dehydrogenase is correlated with the integrity of the cell membrane. The increased LDH level in the CCl_4_-intoxicated group (0.03 IU) mediated cell injury occurred due to the lethal metabolites created by Phase I and II enzyme stimulation. Pre-treated groups with different doses of *Obeth* were shown to significantly reduce the level of LDH equivalent to the control group (**Table S13**) and (**Figure 5I**).

### Histopathological Studies

**Figure 6:**
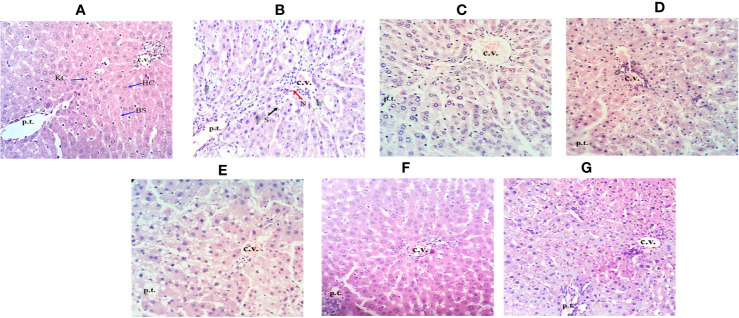
Effect of *Obeth* from *O. bracteata* on histology of liver tissues as assessed by H & E staining. **(A)** Control group (tap water ad libitum) (GI) showing normal lobular architecture with clear portal triad. **(B)** CCl_4_: Olive oil (1:1; 1mL kg^-1^b.wt.) induced necro-inflammatory activity in GII was noted. Treatment of *Obeth* extract was associated with diminution of portal hypertension with increasing dose. **(C)** Silymarin (100 mg kg^-1^b.wt.) + CCl_4_, **(D)** low dose (50 mg kg^-1^b.wt.) + CCl_4_, **(E)** medium dose (100 mg kg^-1^b.wt.) + CCl_4_, **(F)** High dose (200 mg kg^-1^b.wt.) + CCl_4_, **(G)** Negative (only extract 100 mg kg^-1^b.wt. Arrows indicate Kupffer cell (KC), central vein (CV), hepatic cells (HC), blood sinusoids (BS), steatosis (S) (black arrow), and necrosis (N) (red arrow).

The histopathological analysis showed supportive results for the biochemical and serum enzyme markers. The liver of the CCl_4_-treated group showed necrosis, inflammation and loss of hepatocytes with vacuolization when observed between central vein and portal traid area with hematoxylin and eosin staining (**Figure 6B**). In **Figure 6**, compared to other groups, CCl_4_-intoxicated group showed a maximum region of fibrotic markers and collagen localization. *Obeth* and Silymarin treated groups maintained normal liver hepatocyte architecture relative to control group around the central vein area (**Figures 6C–G**).

### Immunohistochemical (IHC) Analysis

**Figure 7:**
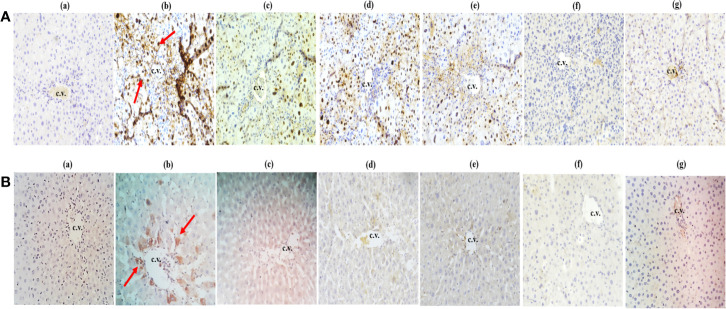
Effect of *Obeth* from *O. bracteata* on expression levels of proteins using immunohistochemical analysis of liver tissues. **(A)** shows cyclin (d) and **(B)** shows p53 antibodies. Arrows showed the increase in expression of cyclin D and p53 proteins in CCl_4_ treated liver samples (Original magnification = 40 x). Expression of these proteins was downregulated when observed in liver samples co-treated with CCl_4_ and *Obeth* (200 mg/kg b.wt.). Red arrow indicates the increase in the expression of protein in the CCl_4_ treated group. (b) Control group (tap water ad libitum) (GI) showing normal lobular architecture with clear portal triad. (b) CCl_4_: Olive oil (1:1; 1mL kg^-1^ b.wt.) induced elevation in the expression level of cyclin D was noted. Treatment of *Obeth* extract was associated with diminution of portal hypertension with increasing dose. (c) Silymarin (100 mg kg^-1^b.wt.) + CCl_4_, (d) low dose (50 mg kg^-1^b.wt.) + CCl_4_, (e) medium dose (100 mg kg^-1^b.wt.) + CCl_4_, (f) High dose (200 mg kg^-1^b.wt.) + CCl_4_, (g) Negative (only extract 100 mg kg^-1^ b.wt.).

Within hepatocytes, over-expression of p53 protein and cyclin-D contribute to hepatocellular carcinoma development. In this experiment, CCl_4_-treated group exhibited an upregulated expression level of cyclin D (**Figure 7A**) and p53 markers (**Figure 7B**) which finally show fibrosis and lobular inflammation whereas, treatment of *Obeth* decreased the CCl_4_ induced p53 and cyclin D protein expression dose-dependently in liver hepatocytes as compared to the toxic group (**Figures 7A**, **c–g** and **7B**, **c–g**).

### Comet Assay

**Figure 8:**
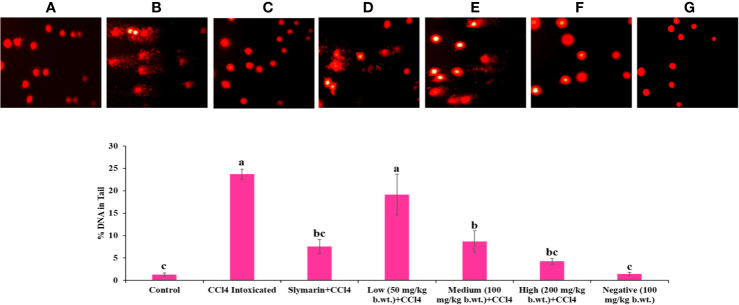
Single cell gel electrophoresis assay was used for determined DNA damage/protect in liver of rats. Results of the comet assay confirm that pretreatment with *Obeth* decreases CCl_4_-induced hepatotoxicity in rats. **(A)** Control group (tap water ad libitum) (GI), **(B)** CCl_4_: Olive oil (1:1; 1mL kg^-1^b.wt.) in GII, **(C)** Silymarin (100 mg kg^-1^b.wt.) + CCl_4_, **(D)** low dose (50 mg kg^-1^b.wt.) + CCl_4_, **(E)** medium dose (100 mg kg^-1^b.wt.) + CCl_4_, **(F)** High dose (200 mg kg^-1^b.wt.) + CCl_4,_ **(G)** Negative (only extract 100 mg kg^-1^b.wt.). Histograms showing densitometric analysis of % of DNA in tail in comet assay. Values are expressed as Mean ± SE at level of significance p ≤ 0.05. Data labels with different letters represent significant difference among them.

As shown in **Figure 8**, larger Comet tail length in CCl_4_ treated groups indicated the higher necrosis, apoptosis and DNA fragmentation in hepatocytes. Whereas, reduced Comet tail length in *Obeth* + CCl_4_ treated groups (100 mg/kg b.wt. and 200 mg/kg b.wt.) showed inhibition of necrosis, apoptosis and DNA fragmentation.

### Western Blotting

#### Protein Expression of p-53, Cox-2, and NfκB

**Figure 9:**
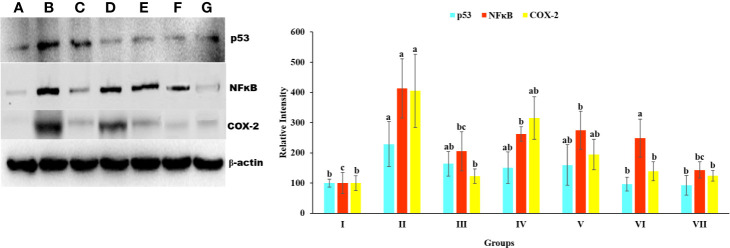
Expression level p53, NFκB, and COX-2 protein in liver homogenate of Wistar rats detected using Western blotting. **(A)** Control group (tap water ad libitum) (GI), **(B)** CCl_4_: Olive oil (1:1; 1mL kg^-1^b.wt.) induced elevation in the expression level of p53 was noted. **(C)** Silymarin (100 mg kg^-1^b.wt.) + CCl_4_, **(D)** low dose (50 mg kg^-1^b.wt.) + CCl_4_, **(E)** medium dose (100 mg kg^-1^b.wt.) + CCl_4_, **(F)** High dose (200 mg kg^-1^b.wt.) + CCl_4,_ **(G)** Negative (only extract 100 mg kg^-1^ b/wt.). Histograms showing densitometric analysis of p53, NFκB, and COX-2 protein bands using western blotting. Band density was measured and normalized to that of β-actin. Values are expressed as mean ± SE at level of significance p ≤ 0.05. Data labels with different letters represent significant difference among them.

To explore the relationship between p53, COX-2, and NfκB and the hepatoprotective effects of *Obeth* on toxicity induced by CCl_4_, we studied protein expression using western blotting. As shown in **Figure 9**. there was a significant increased (*p* < 0.05) in p53, Cox-2, and NfκB protein expression in CCl_4_ treated group as compared to the untreated control group. However, there was a dose-dependent decrease in the expression of proteins on administration of *Obeth* with CCl_4_ treated groups as compared to only CCl_4_ intoxicated group.

### Phytochemical Analysis by HPLC Analysis

**Figure 10:**
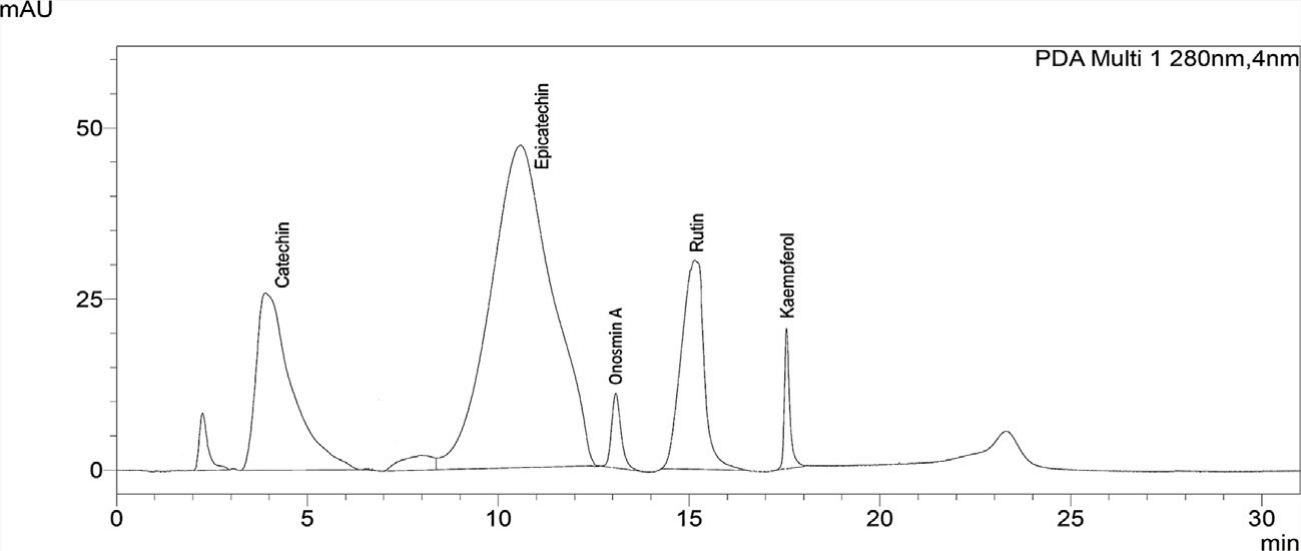
Chromatogram of *Obeth* of *O. bracteata* showing presence of polyphenols as identified using HPLC analysis by reference compound.

The phytochemical analysis by HPLC exhibited the occurrence of catechin, epicatechin, Onosmin A, rutin and kaempferol as major phytoconstituents in *Obeth* (**Figure 10**, **Table 2**).

**Table 2:** Amount (mg/L) of different phytochemical quantified by HPLC in different *Obeth* of *O. bracteata*.

Compounds (*Obeth*)	Concentration (mg/L)
Gallic acid	–
Catechin	203.29
Chlorogenic acid	–
Epicatechin	844.04
Caffeic acid	–
Umbelliferone	–
Rutin	49.84
Ellagic acid	–
Quercetin Onosmin A	– 21.82
Kaempferol	34.82
Coumaric acid	–

## Discussion

The active polyphenols present in the plants act as scavengers of ROS, DNA protective agents and help to maintain the expression of genes/proteins that break down toxins and keep cellular redox homeostasis (Liu et al., 2018). These polyphenols have the antioxidant, antimutagenic and chemopreventive properties that might help to protect and conserve the constancy of genome (Mehta et al., 2010; Rodríguez-García et al., 2019). Previous studies have reported the potential of *Onosma* genus as antioxidant compounds and their use in averting numerous pharmacological situations (Naz et al., 2006; Ebrahimzadeh et al., 2010; Ahmad et al., 2013; Imran et al., 2018; Farooq et al., 2019).

In the current study, we investigated the antioxidant, antimutagenic, genoprotective, and hepatoprotective properties of *Obeth* from *Onosma bracteata* along with evaluation of the expression of proteins involved in the inflammation and apoptotic induction in *in vivo* studies using male Wistar rats. In antioxidant assays, *Obeth* had shown potential to scavenge free radicals, namely in SRS and LPO assays. *Obeth* showed strong radical scavenging activity with EC_50_ (115.14 µg/mL) whereas LPO inhibition activity with EC_50_ (199.33 µg/mL) (**Tables S1** and **S2**). Radical scavenging potential of *Obeth* might be correlated to the nature of phenolics, contributing to their electron transfer/hydrogen donating ability.

Plants contain natural antioxidants, primarily flavonoids, which are being explored to inhibit various diseases *viz*. chronic inflammation and cancer (Surh, 2003). *Onosma* genus plants are enriched with bioactive components *viz*. flavonoids, alkannin, and shikonin responsible for wound healing, analgesic and its antibacterial actions (Kumar et al., 2013). The HPLC analysis of *Obeth* revealed the existence of different phytoconstituents *viz*., kaempferol, Onosmin A, catechin and epicatechin. Numerous reports have shown that *Onosma* genus and its isolated compounds are responsible for excellent antioxidant activities (Riedl et al., 2004; Kandemir and Türkmen, 2008; Menghani et al., 2011). The ethyl acetate, methanol and water extracts from *Onosma heterophylla* Griseb. have an appreciable amount of kaempferol, caffeic acid, o-Coumaric acid, rutin and rosmarinic acid which are responsible for antioxidant activity (Ozer et al., 2018). Saravanakumar et al., 2019 also reported that the methanol extract of *Onosma isaurica* Boiss. & Heldr. and *Onosma bracteosa* Hausskn. & Bornm. showed effective free radical scavenging ability due to the presence of the phytoconstituents. This plant has been widely used because of its medicinal properties.

In Ames assay, the antimutagenic potential of *Obeth* against sodium azide, 4-nitro-o-phenylenediamine and 2- aminofluorene mutagens was evaluated against two *Salmonella typhimurium* strains, i.e., TA100 and TA98. The study showed that *Obeth* effectively modulated the mutagenicity of direct-acting base-pair mutagen, sodium azide with 82.30% inhibition at 250 μg/0.1 mL/plate (**Table S3**) in TA 100 while it showed moderate antimutagenic effect against 4NPD with 62.85% inhibition at the same concentration in TA98 tester strain. *Obeth* exhibited antimutagenicity against 2AF (S9 dependent) in both the tester strains. Kaur et al., 2001 reported that phenolic fractions isolated from *Terminalia arjuna* showed mutagen specificity *via* decreasing the frameshift mutagen NPD whereas it failed in the prevention of sodium azide (base pair substitution)-induced his^+^ revertants. Kaur et al., 2016 described that ethyl acetate fraction of *Cassia fistula* (CaFE) showed strong antimutagenic and antioxidant properties due to the presence of polyphenolic compounds (umbelliferone, catechin and epicatechin). *Obeth* also demonstrated the potential to protect plasmid DNA against free radicals of Fenton’s reagent in plasmid DNA nicking assay. *Obeth* at 100 µg/mL concentration efficiently retained DNA’s supercoiled form.

In *in vivo* studies, serum analysis showed that the oxidative stress serum markers (SGOT, SGPT, ALP, creatinine and direct bilirubin) were significantly increased, following the dosage of CCl_4_ to rats (Group II) (**Tables S5**, **S6**, and **S8**). The potential to produce albumin proteins (which function as transport molecules) vanished in damaged hepatocytes, and the capacity to make inflammatory proteins increased. CCl_4_ generated free radicals (trichloromethyl and peroxytrichloromethyl) that induced damage in hepatocytes resulting in leakage of cytoplasmic SGPT and SGOT into the bloodstream (Shah et al., 2015). *Obeth* ameliorated the toxic effects of CCl_4_ by enhancing the activity of antioxidant molecules in the liver. *Obeth* significantly decreased the damage caused by CCl_4_ (Group II) in cellular TBARS. A decrease in GSH enzymes and protein levels was observed in the CCl_4_ treated group (Group II), while an increase in these markers was observed following *Obeth* treatment (**Table S9**). Sabir et al. (2017) reported in their study that aqueous extracts of *Zanthoxylum armatum* DC. (syn. *Zanthoxylum alatum* Roxb.) restored the level of glutathione and showed hepatoprotective property against paracetamol-induced hepatic damage in albino mice which decreased the hepatic glutathione level. The activation of lipid hydroperoxides, Cytochrome P_450_, P_420_, and b5 were observed in CCl_4_ treated group whereas *Obeth* treatment declined the level of these markers (**Table S10**). The values of the above parameters in the *Obeth*-treated group were the same as of the control (GroupI) which reflects a high safety potential of *Obeth*. CCl_4_ treated group showed decline GST levels as compared to control but in a dose-dependent manner whereas *Obeth* treated groups exhibited restored level of GST. Allocati et al., 2018 reported that GSTs can protect DNA against oxidative stress and eliminate toxic molecules that can led to mutations or carcinogenesis. Laouar et al., 2017 reported that treatment of CCl_4_ lead to significantly reduced levels of hepatic GSH, GPx, and GST activities than control group in Wistar rats, whereas aqueous extract of *Juniperus phoenicea* L. berries treatment significantly restored the levels of these enzymes. The Group II showed a significant increase in the amount of NADPH cytochrome b5 and NADH cytochrome 450, whereas, *Obeth* treated groups exhibited a dose-dependent decline in the level of these enzymes (**Table S11**). The CCl_4_ treatment generates CCl_3_^•^ radicals which not only damaged DNA but also caused necrosis and apoptosis as evidenced from increase in Comet tail length in CCl_4_ treated group whereas in case of *Obeth* -treated groups, the reduced Comet tail length showed the DNA protective activity of *Obeth* in a dose-dependent manner (**Figure 8**). Damage of cells by CCl_3_^•^ free radical (toxic) formed by the reduction of CCl_4_, in hepatocytes has also been reported Brai et al. (2014) and Josan et al. (2015). The previous report from Younis et al., 2018 showed that CCl_4_ treated rats showed a high degree of DNA damage and remarkable changes in (comet, head, tail) length and %DNA in the tail, while methanolic extract of *Fraxinus xanthoxyloides* (G.Don) Wall. ex A.DC. ameliorates the effect of CCl_4_ similar to that of control group.

Histopathological studies showed that *Obeth* was itself non-toxic and showed close histological similarity to the control group and displayed the same histological arrangement with no sign of inflammation (**Figure 6G**). In contrast, CCl_4_ treated group displayed steatosis, necrosis, inflammation in blood sinusoids and vacuolization (**Figure 6B**). However, pretreatment of *Obeth* followed by CCl_4_ treatment effectively restored the regular hepatocytes’ architecture and also reduces the vacuoles formation between the central vein and portal triad indicating hepatoprotective potential of *Obeth*, as shown in **Figures 6D–F**. Earlier, Josan et al. (2015) reported that CCl_4_ treated Sprague Dawley rats showed necrosis of hepatocytes nearby central vein and increased the level of alanine transaminase (ALT) whereas control group showed normal architecture of hepatocytes. Ganaie et al., 2015 also reported that dose-dependent treatment of ethanolic extract of *Rumex vesicarius* L. showed a restored pattern of histological architecture, while silymarin treatment followed by CCl_4_-intoxication in Wistar rats showed less disarrangement of hepatocytes. The results of the Immunohistochemistry (IHC) studies showed that p53 protein and Cyclin D expressions in the liver tissues were maximum in the CCl_4_ intoxicated group. Upregulated expression of pro-apoptotic p53 in CCl_4_ treated group might be formed in response to hepatocyte inflammation and the induction of necrosis in liver cells (**Figures 7A, B**). Similar results were observed in western blotting studies showing p53 upregulation in the CCl_4_ group as compared with the control group. The tumor suppressor gene p53 is upregulated in response to DNA damage, and its upregulated expression is only found in cells that show mutant forms (Aly et al., 2019). The upregulated expression of Cyclin D protein in the CCl_4_ intoxicated group also highlighted the mutation in hepatocytes while *Obeth* treatment showed the downregulation of Cyclin D exhibiting its protective effects against CCl_4_ intoxication. Cyclin D has been described to have the main role in cell progression through the G1/S phase of the cell cycle, and its deregulation can lead to tumorigenesis (Ying et al., 2008; Chibazakura et al., 2011; Gao et al., 2016).

**Figure 11:**
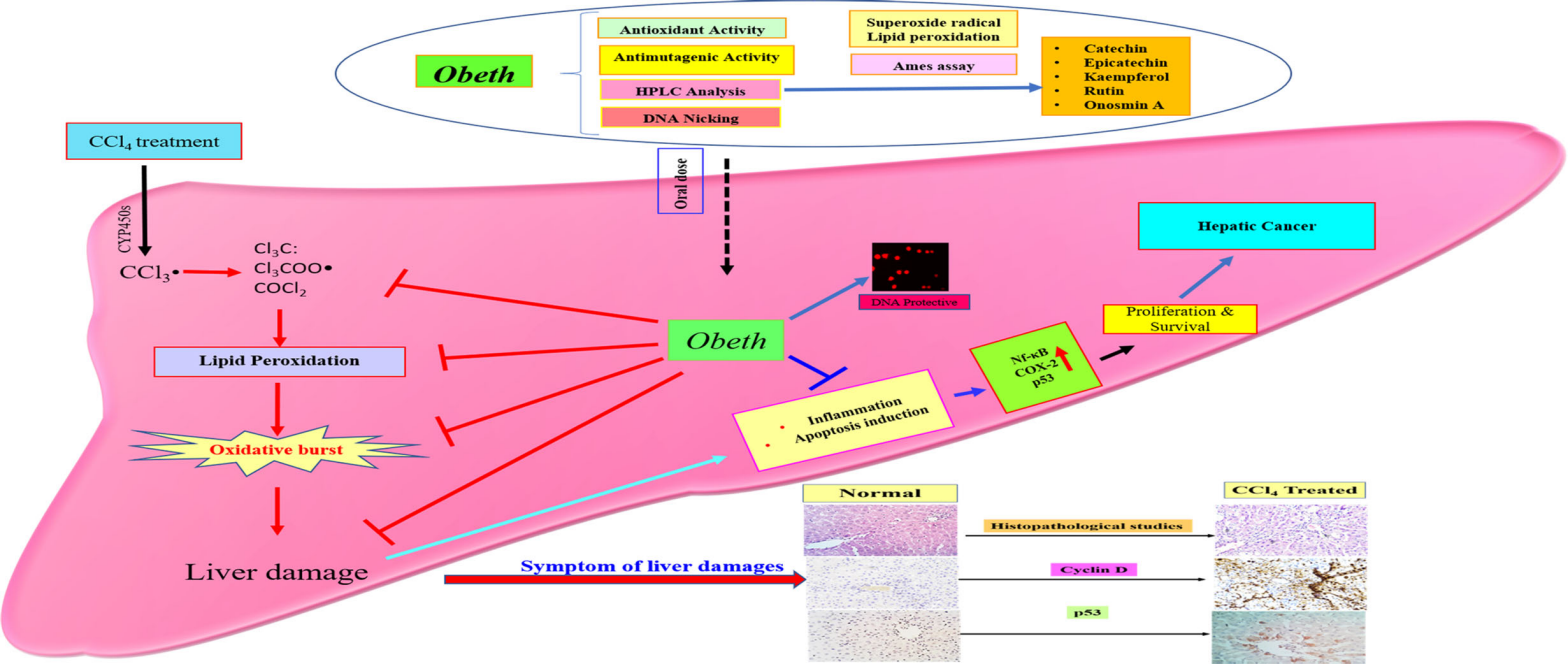
Schematic proposed mechanisms for hepatoprotective property of *Obeth* from *O. bracteata*. *Obeth* phytoconstituents ameliorated hepatic oxidative stress and suppressed inflammation, apoptosis, and DNA damage in CCl_4_-injured hepatocytes.

The expression of p53, COX-2, and NF-κB was upregulated due to cellular stress, inflammation and mutation in cellular DNA (Banerjee et al., 2002; Cha and DeMatteo, 2005). Hepatic cancer can be controlled by the suppression of COX-2, NF-κB and p53 which might offer an effective control approach. The investigation of the effects of *Obeth* towards stimulated forms of p53, cyclin-D, COX-2 and NF- κB proteins was performed. The expression levels of COX-2 and NF-κB proteins were upregulated after CCl_4_ administration (**Figure 9**) highlighting the production of mediator cytokines which facilitate tumor growth *via* inhibiting apoptosis and promoting cell proliferation (Brücher et al., 2019; Lerdwanangkun et al., 2019). Eltahir et al. (2020) reported that CCl_4_ treated Wistar rats showed significantly high expression levels of NF- κB and COX-2 while *Boswellia serrata* Roxb. (BS) gum resin treatment followed by CCl_4_ significantly restored the level of NF- κB and COX-2 expression due to the presences of a natural compound that exhibited anti-inflammatory effects by targeting multiple pathways. The stressed cells promoted the overactivation of signaling proteins or inflammatory cytokines including, IL-6, TNFα, and IL-1β that stimulate the overproduction of IL-6 in Kupffer cells leading to the enhanced proliferation of hepatocytes, some of which harbor oncogenic mutations (Sun and Karin, 2008). *Obeth* has efficient antioxidant properties that protect the hepatocytes against any damage by inducing antioxidative mechanisms. Previous reports have proved the connotation among natural compounds isolated from plants and cancer inhibition *via* antioxidant properties (Seufi et al., 2009). In the present study, it was found that the *Obeth* contains catechin, epicatechin, rutin, kaempferol, and Onosmin A (**Figure 10**, **Table 2**). All these compounds have antioxidant potential are known to ascertain their remedial effects by scavenging the free radical species due to the presence of H donating hydroxyl groups on the different polyphenolic rings of these molecules stabilize the negative charge on the different reactive species and hence, nullify their damaging consequences (Kaur P. et al., 2019). One such study showed catechin to regulate the expression of NF-κB and Cox-2 by scavenging the ROS and hence delivering the anti-inflammatory and antioxidant effects (Chu et al., 2017). Similarly, rutin has also proven as an effective hepatoprotective agent against CCl_4_ induced Wistar rat liver damage by altering the expression of IL-6/STAT3 pathway genes expression due to its antioxidative, anti-inflammatory and anti-apoptotic properties (Hafez et al., 2015). On the other hand, epicatechin has the potential to inhibit Sprague-Dawley rat liver inflammation *via* NF*κ*B signaling pathway initiated by HSP60 (Huang et al., 2019). Zang et al., 2018 reported that soybean leaves and its isolated compound kaempferol galactoside and kaempferol have the ability to reduce serum parameters (serum aspartate aminotransferase and serum alanine aminotransferase) increased by CCl_4_ intoxication. In addition to this, all these compounds were also found to ameliorate the lipid peroxidation and regulate the levels of different liver enzymes (Sinha et al., 2012; Khan et al., 2012; Liu et al., 2015; Wang et al., 2015). The polyphenolics such as catechin, caffeine, epicatechin (EC), epigallocatechin gallate (EGCG), and epicatechin gallate (ECG) showed preventive effects of Liubao Tea against CCl_4_ -induced liver injury in Kunming mice, comparable to silymarin (Pan et al., 2018). Ahmad et al., 2005, reported that Onosmin A isolated from *Onosma hispida* Wall. ex G.Don has potential to inhibit lipogenase enzyme in a concentration-dependent manner. It can be stated that the chemical association of phytochemicals, presumable flavonoids in *Obeth* from *O. bracteata* have protective effects on liver damage *via* suppression of CCl_4_-induced oxidative stress (**Figure 11**). This study reported that antioxidant, antimutagenic, DNA protective, and anti-inflammatory properties of *Obeth* are correlated with the phytoconstituents present in and have also been shown to have modulatory impact on pro-carcinogenic proteins p53, NF-κB, and COX-2 for the first time.

## Conclusions

In the current study, *Obeth* of *O. bracteata* has shown significant antioxidant, genoprotective, antimutagenic and hepatoprotective properties. As revealed by HPLC investigations, *Obeth* harbors catechin, epicatechin, rutin, Onosmin A and kaempferol as major phytoconstituents. *Obeth* exerted its protective potential *via* its radical scavenging and DNA protective abilities, minimizing oxidative stress through induction of antioxidative enzymes and ameliorating the hepatic changes triggered by CCl_4_. The modulation of crucial proteins such as p53, cyclin D, Cox-2, and NF-κB demonstrated potential of *Obeth* as an effective chemotherapeutic agent against hepatic cancer.

## Data Availability Statement

The raw data supporting the conclusions of this article will be made available by the authors, without undue reservation, to any qualified researcher.

## Ethics Statement

The animal study was reviewed and approved by Committee for the Purpose of Control and Supervision of Experiments on Animals (CPCSE), Ministry of Environment and Forestry, Government of India, and authorized by Institutional Animal Ethical Committee (IAEC), Guru Nanak Dev University, Amritsar, India (approval number 226/CPCSEA/2014/06).

## Author Contributions

AK: formal analysis, investigation, methodology, data curation, and writing—original draft. HT and KS: helped to revise the manuscript, reviewing, and editing. VK: intellectual contribution and reviewing manuscript. KP: formal analysis. SJ: conceptualization and project administration. SK: conceptualization, supervision, project administration, reviewing, editing, and resources. All authors contributed to the article and approved the submitted version.

## Conflict of Interest

The authors declare that the research was conducted in the absence of any commercial or financial relationships that could be construed as a potential conflict of interest.

## Abbreviations

2AA, 2-aminoanthracene; 2-AF, 2-aminofluorene; AA, Ascorbic acid; BC, bacterial culture; b.wt., body weight; CCl_4_, carbon tetrachloride; COX-2, cyclooxygenase-2; CPCSEA, committee for the Purpose of Control and Supervision of Experiments on Animals; ANOVA, analysis of variance. CYP, cytochrome P_450_; df, Degree of freedom; EDTA, ethylenediamine tetraacetic acid; ECL, Enhanced chemiluminescence; FeCl_3_, Ferric chloride; GSH, reduced glutathione; GSSG, oxidized glutathione; HPLC, high-performance liquid chromatography; i.p, intraperitoneal; MDA, malondialdehyde; NBT, nitro blue tetrazolium*;* NF-κB, nuclear factor-kappaB; NPD, 4- Nitro-o-phenylenediamine; PAGE, polyacrylamide gel electrophoresis; PBS, phosphate buffer saline; PMS, Phenazine methosulfate; PVDF, polyvinylidene fluoride; ROS, reactive oxygen species; rpm, revolutions per minute; SA, sodium azide; SDS, sodium dodecyl sulphate; TA, top agar; TCA, trichloroacetic acid; WB, western blotting.
