# Supplementary material for: Antioxidant Phytoconstituents From Onosma bracteata Wall. (Boraginaceae) Ameliorate the CCl4 Induced Hepatic Damage: In Vivo Study in Male Wistar Rats
Source: Front Pharmacol. 2020 Aug 21;11:1301. doi: 10.3389/fphar.2020.01301 (PMC7472603; doi:10.3389/fphar.2020.01301)
Supplement: Supplementary file 1 [file DataSheet_1.docx]

**TABLE S1. Antioxidant activity of *Obeth* extract of *O. bracteata* in** **Superoxide radical scavenging assay**

| **Conc**  **(μg/mL)** | **Scavenging (%)** | |
| --- | --- | --- |
|  | **Rutin** | ***Obeth*** |
| **25** | 39.36± 2.60^c^ | 16.72± 1.35^d^ |
| **50** | 43.91± 2.93^c^ | 24.84± 0.68^d^ |
| **100** | 74.29± 1.09^b^ | 51.29± 1.91^c^ |
| **200** | 77.74± 1.45^b^ | 61.74± 2.25^b^ |
| **400** | 90.16± 1.07^a^ | 78.96± 2.59^a^ |
| **EC_50_ (μg/mL)** | 46.18 | 115.14 |
| **Regression equation** | y = 19.53ln(x) - 24.88 | y = 23.28ln(x) - 60.50 |
| **R^2^** | 0.924 | 0.977 |
| **F-ratio** | 125.20* | 187.19* |
| **HSD** | 9.25 | 8.77 |

**Values are represented as Mean ± SE, *level of significance p≤0.05. Means with different superscripts represent significant difference among them in all groups.**

**TABLE S2. Antioxidant activity of *Obeth* extract of *O. bracteata* in** **Lipid peroxidation assay.**

| **Conc (μg/mL)** | **Scavenging (%)** | |
| --- | --- | --- |
|  | **Rutin** | ***Obeth*** |
| **25** | 19.78±1.04^d^ | 11.62± 1.36^e^ |
| **50** | 39.45 ± 2.09^c^ | 17.84± 1.51^d^ |
| **100** | 48.48 ± 1.63^bc^ | 30.55± 0.81^c^ |
| **200** | 56.55± 2.24^b^ | 52.19± 0.63^b^ |
| **400** | 72.78± 3.39^a^ | 66.29± 1.66^a^ |
| **EC_50_ (μg/mL)** | 115.68 | 199.33 |
| **Regression equation** | y = 17.75ln(x) - 34.37 | y = 20.73ln(x) - 59.76 |
| **R^2^** | 0.974 | 0.967 |
| **F-ratio** | 78.69* | 331.68* |
| **HSD** | 10.33 | 5.89 |

**Values are represented as Mean ± SE, *level of significance p≤0.05. Means with different superscripts represent significant difference among them in all groups.**

**TABLE S3. Antimutagenic effect of *Obeth* of *O. bracteata* in Ames assay with Two-way ANOVA analysis.**

| **Expression mode** | **Regression equation** | **(r)** | **EC_50_ (µg/0.1 mL/plate)** | **Difference between co-incubation and pre- incubation experimentation**  **(df=1)** | **Difference between *Obeth* doses or concentrations applied (df=1)** | **Interaction among experimentation mode and doses applied (df=4)** |
| --- | --- | --- | --- | --- | --- | --- |
| TA98 (without S9) co-incubation | y = 15.38ln(x) - 23.14 | 0.973 | 116.04 | 21.72* | 218.62* | 2.39 |
| TA98 (without S9) pre-incubation | y = 12.01ln(x) - 0.63 | 0.962 | 67.69 |  |  |  |
| TA98 (with S9) co-incubation | y = 16.17ln(x) - 4.74 | 0.987 | 53.41 | 52.22* | 330.48 | 6.79 |
| TA98 (with S9) pre-incubation | y = 9.39ln(x) + 12.62 | 0.953 | 29.51 |  |  |  |
| TA100 (without S9) co-incubation | y = 14.59ln(x) + 4.01 | 0.981 | 42.68 | 20.89* | 338.58* | 1.40* |
| TA100 (without S9) pre-incubation | y = 14.59ln(x) + 4.01 | 0.981 | 23.37 |  |  |  |
| TA100 (with S9) co-incubation | y = 15.38ln(x) - 23.14 | 0.990 | 16.48 | 11.81* | 323.54* | 1.08* |
| TA100 (with S9) pre-incubation | y = 13.74ln(x) + 17.70 | 0.979 | 10.48 |  |  |  |

* represent level of significance p≤ 0.0.5 for all sample. Degree of freedom= df.

**TABLE S4. Percent change in body weight of male Wistar rats after treatment for 21 days.**

|  | **Treatments** | **Percentage change in body weight after 3 weeks ± S.E**  **(Decrease in fold)** |
| --- | --- | --- |
| **Group I** | Control (tap water *ad libitum*) | 1 ± 0^a^ |
| **Group II** | CCl_4_(1mL/kg^-1^b.wt.) | 0.89 + 5.87^b^ |
| **Group III** | CCl_4_ + silymarin (100 mg kg^-1^b.wt.) | 0.98 ± 2.62^a^ |
| **Group IV** | *Obeth* (200 mg kg^-1^ b.wt.) | 1 ± 2.24^a^ |
| **Group V** | CCl_4_ + *Obeth* (50 mg kg^-1^b.wt.) | 0.95 ± 0.83^ab^ |
| **Group VI** | CCl_4_ + *Obeth* (100 mg kg^-1^b.wt.) | 0.98 ± 1.54^a^ |
| **Group VII** | CCl_4_ + *Obeth* (200 mg kg^-1^b.wt.) | 1 ± 2.98^a^ |
|  | HSD | 0.05 |
|  | F-ratio | 8.26* |

**Values are represented as Mean ± SE (for n = 6 in each group), *level of significance p≤0.05. Means with different superscripts represent significant difference among them in all groups.**

**TABLE S5. The effect of *Obeth* on CCl_4_-induced alterations in serum hepatic enzymes including aspartate aminotransferase (AST), alanine aminotransferase (ALT) and aspartate alkaline phosphatase (ALP).**

| **Groups** | | **Doses (mg kg^-1^bw)** | **Serum enzyme activities (U/L) (Mean ± SE)** | | |
| --- | --- | --- | --- | --- | --- |
|  |  |  | **SGOT ALP SGPT** | | |
| **I** | Control (tap water *ad libitum*) | | 95.67 ± 28.15^d^ | 158.33 ± 16.86^cd^ | 55.56 ± 9.49^bc^ |
| **II** | CCl_4_(1mL/kg^-1^b.wt.) | | 351.45 ± 21.01^a^ | 529.16 ± 56.74^a^ | 133.86 ± 29.88^ab^ |
| **III** | CCl_4_ + silymarin (100 mg kg^-1^b.wt.) | | 285.82 ± 43.64^ab^ | 484.16 ± 40.49^ab^ | 85.01 ± 7.05^abc^ |
| **IV** | *Obeth* (200 mg kg^-1^ b.wt.) | | 198.98 ± 20.09^bcd^ | 120.83 ± 14.33^d^ | 54.87 ± 9.30^c^ |
| **V** | CCl_4_ + *Obeth* (50 mg kg^-1^b.wt.) | | 259.00 ± 21.16^abc^ | 467.66 ± 69.91^ab^ | 124.05 ± 7.98^ab^ |
| **VI** | CCl_4_ + *Obeth* (100 mg kg^-1^b.wt.) | | 173.05 ± 11.51^cd^ | 324.16 ± 19.72^bc^ | 87.07 ± 22.55^abc^ |
| **VII** | CCl_4_ + *Obeth* (200 mg kg^-1^b.wt.) | | 168.43 ± 3.77^cd^ | 146.83 ± 5.27^d^ | 81.46 ± 3.80^abc^ |
|  | | HSD | 107.16 | 171.96 | 68.81 |
|  | | F-ratio | 12.47* | 20.77* | 3.86* |

**Values are represented as Mean ± SE (for n = 6 in each group), *level of significance p≤0.05. Means with different superscripts represent significant difference among them in all groups.**

**TABLE S6. The effect of *Obeth* on CCl_4_-induced alterations in serum hepatic enzymes including direct bilirubin and total bilirubin.**

| **Groups** | **Doses (mg kg^-1^bw)** | **Serum enzyme activities (mg/dL) (Mean ± SE)** | |
| --- | --- | --- | --- |
|  |  | **Direct bilirubin Total bilirubin** | |
| **I** | Control (tap water *ad libitum*) | 0.07 ± 0.02^c^ | 0.13 ± 0.02^c^ |
| **II** | CCl_4_(1mL/kg^-1^bw) | 0.18 ± 0.03^abc^ | 0.39 ± 0.03^a^ |
| **III** | CCl_4_ + silymarin (100 mg kg^-1^b.wt.) | 0.20 ± 0.05^a^ | 0.24 ± 0.03^bc^ |
| **IV** | *Obeth* (200 mg kg^-1^ bw) | 0.10 ± 0.01^bc^ | 0.17 ± 0.02^bc^ |
| **V** | CCl_4_ + *Obeth* (50 mg kg^-1^b.wt.) | 0.18 ± 0.03^ab^ | 0.28 ± 0.03^ab^ |
| **VI** | CCl_4_ + *Obeth* (100 mg kg^-1^b.wt.) | 0.17 ± 0.02^abc^ | 0.28 ± 0.04^ab^ |
| **VII** | CCl_4_ + *Obeth* (200 mg kg^-1^b.wt.) | 0.11 ± 0.01^bc^ | 0.25 ± 0.04^bc^ |
|  | HSD | 0.12 | 0.13 |
|  | F-ratio | 3.34* | 7.42* |

**Values are represented as Mean ± SE (for n = 6 in each group), *level of significance p≤0.05. Means with different superscripts represent significant difference among them in all groups.**

**TABLE S7. The effect of *Obeth* on CCl_4_-induced alterations in serum hepatic enzymes including albumin and total protein.**

| **Groups** | **Doses (mg kg^-1^bw)** | **Serum enzyme activities (g/dL) (Mean ± SE)** | | |
| --- | --- | --- | --- | --- |
|  |  | **Albumin Total protein** | | |
| **I** | Control (tap water *ad libitum*) | | 3.8 ± 0.37^a^ | 7.97 ± 0.14^a^ |
| **II** | CCl_4_(1mL/kg^-1^b.wt.) | | 2.15 ± 0.3^c^ | 5.93 ± 0.34^d^ |
| **III** | CCl_4_ + silymarin (100 mg kg^-1^b.wt.) | | 3.26 ± 0.19^ab^ | 6.48 ± 0.08^c^ |
| **IV** | *Obeth* (200 mg kg^-1^ b.wt.) | | 3.77 ± 0.42^a^ | 6.42 ± 0.05^cd^ |
| **V** | CCl_4_ + *Obeth* (50 mg kg^-1^b.wt.) | | 2.47 ± 0.21^bc^ | 5.95 ± 0.13^d^ |
| **VI** | CCl_4_ + *Obeth* (100 mg kg^-1^b.wt.) | | 3.2 ± 0.20^ab^ | 6.43 ± 0.23^cd^ |
| **VII** | CCl_4_ + *Obeth* (200 mg kg^-1^b.wt.) | | 3.61 ± 0.08^a^ | 7.38 ± 0.09^b^ |
|  | HSD | 0.99 | | 0.80 |
|  | F-ratio | 8.28* | | 17.31* |

**Values are represented as Mean ± SE (for n = 6 in each group), *level of significance p≤0.05. Means with different superscripts represent significant difference among them in all groups.**

**TABLE S8. Effect of *Obeth* and CCl_4_ alone and in combination on various serum parameters.**

| **Groups** | **Doses (mg kg^-1^bw)** | **Urea**  **(mg per dL)**  **± SE** | | **Creatinine**  **(mg per dL)**  **± SE** | **Cholesterol CHOL**  **(mg per dL) ± SE** | **Triglycerides**  **TG**  **(mmol per L) ± SE** |
| --- | --- | --- | --- | --- | --- | --- |
| **I** | Control (tap water *ad libitum*) | | 49.28 ± 6.50^c^ | 0.74 ± 0.04^c^ | 35 ± 2.55^a^ | 35 ± 2.55^c^ |
| **II** | CCl_4_(1mL/kg^-1^b.wt.) | | 96.63 ± 12.17^a^ | 1.87 ± 0.28^a^ | 65.17 ± 6.82^ab^ | 77.5 ± 5.91^a^ |
| **III** | CCl_4_ + silymarin (100 mg kg^-1^b.wt.) | | 70.27 ± 2.92^abc^ | 1.46 ± 0.17^ab^ | 44.3 ± 2.05^bc^ | 57.5 ± 4.01^abc^ |
| **IV** | *Obeth* (200 mg kg^-1^ b.wt.) | | 59.72 ± 2.89^c^ | 0.77 ± 0.01^bc^ | 45 ± 1.22^bc^ | 49.83 ± 2.09^bc^ |
| **V** | CCl_4_ + *Obeth* (50 mg kg^-1^b.wt.) | | 86.77 ± 3.46^ab^ | 1.59 ± 0.14^a^ | 79.33 ± 3.92^a^ | 69.83 ± 6.30^ab^ |
| **VI** | CCl_4_ + *Obeth* (100 mg kg^-1^b.wt.) | | 72.87 ± 3.50^abc^ | 0.89 ± 0.1^bc^ | 58.67 ± 5.22^ab^ | 59.17 ± 9.23^ab^ |
| **VII** | CCl_4_ + *Obeth* (200 mg kg^-1^b.wt.) | | 68.43 ± 4.58^bc^ | 1.31 ± 0.17^abc^ | 72 ± 7.70^a^ | 51.33 ± 1.89^bc^ |
|  | HSD | 26.48 | | 0.70 | 21.12 | 22.99 |
|  | F-ratio | 7.01* | | 7.72* | 11.56* | 7.14* |

**Values are represented as Mean ± SE (for n = 6 in each group), *level of significance p≤0.05. Means with different superscripts represent significant difference among them in all groups.**

**TABLE S9. Effect of *Obeth* and CCl_4_ alone and in combination on various biochemical parameters.**

| **Groups** | | **Doses (mg kg^-1^bw)** | **TBARS (nM**  **MDA equivalent/**  **g of tissue) ± SE** | **Lipid hydro-**  **peroxides**  **(nM H_2_O_2_**  **equivalent/g**  **of tissue) ± SE** | **Protein content**  **(mg/g of tissue)**  **± SE** | **Reduced**  **glutathione**  **content (****µmol**  **of SH content/**  **g of tissue) ± SE** |
| --- | --- | --- | --- | --- | --- | --- |
| **I** | Control (tap water *ad libitum*) | | 0.52 ± 0.14^c^ | 9.43 ± 0.79^c^ | 52.74 ± 11.37^a^ | 231.82 ± 35.38^a^ |
| **II** | CCl_4_(1mL/kg^-1^b.wt.) | | 2.00 ± 0.59^a^ | 23.24 ± 2.54^a^ | 24.89 ± 4.16^c^ | 85.99 ± 9.57^c^ |
| **III** | CCl_4_ + silymarin (100 mg kg^-1^b.wt.) | | 1.32 ± 0.58^abc^ | 11.94 ± 2.63^bc^ | 48.04 ± 3.05^a^ | 209.75 ± 14.71^a^ |
| **IV** | *Obeth* (200 mg kg^-1^ b.wt.) | | 0.67 ± 0.33^bc^ | 10.71 ± 1.35^c^ | 53.38 ± 4.21^a^ | 224.59 ± 18.67^a^ |
| **V** | CCl_4_ + *Obeth* (50 mg kg^-1^b.wt.) | | 1.88 ± 0.15^ab^ | 21.48 ± 1.82^a^ | 27.98 ± 4.69^bc^ | 131.44 ± 26.54^bc^ |
| **VI** | CCl_4_ + *Obeth* (100 mg kg^-1^b.wt.) | | 0.91 ± 0.36^abc^ | 17.20 ± 2.9^ab^ | 36.38 ± 6.60^abc^ | 152.11 ± 27.09^ab^ |
| **VII** | CCl_4_ + *Obeth* (200 mg kg^-1^b.wt.) | | 0.64 ± 0.39^bc^ | 13.12 ± 1.35^bc^ | 45.25 ± 3.44^ab^ | 197.14 ± 15.27^ab^ |
|  | | HSD | 1.26 | 6.40 | 29.54 | 108.95 |
|  | | F-ratio | 4.55* | 14.03* | 3.02* | 4.77* |

**Values are represented as Mean ± SE (for n = 6 in each group), *level of significance p≤0.05. Means with different superscripts represent significant difference among them in all groups.**

**TABLE S10. Effect of *Obeth* on cytochrome P450, cytochrome P420 and cytochrome b5 content among different treatment groups.**

| **Groups** | | **Doses (mg kg^-1^bw)** | **Cytochrome P450**  **content (µmol/ mg protein)** | **Cytochrome P420**  **content (µmol/ mg**  **protein)** | **Cytochrome b5**  **content (µmol/ mg protein)** |
| --- | --- | --- | --- | --- | --- |
| **I** | Control (tap water *ad libitum*) | | 27.7 ± 4.9^ab^ | 174.95 ± 11.45^d^ | 29.34 ± 2.27^d^ |
| **II** | CCl_4_(1mL/kg^-1^b.wt.) | | 45.90 ± 5.07^a^ | 329.18 ± 26.63^a^ | 98.32 ± 5.74^a^ |
| **III** | CCl_4_ + silymarin (100 mg kg^-1^b.wt.) | | 22.76 ± 5.29^b^ | 208.85 ± 3.61^cd^ | 36.38 ± 3.16^cd^ |
| **IV** | *Obeth* (200 mg kg^-1^ b.wt.) | | 27.20 ± 2.9^ab^ | 202.51 ± 4.51^d^ | 35.73 ± 2.56^cd^ |
| **V** | CCl_4_ + *Obeth* (50 mg kg^-1^b.wt.) | | 34.52 ± 6.89^ab^ | 293.71 ± 4.01^ab^ | 67.82 ± 4.93^b^ |
| **VI** | CCl_4_ + *Obeth* (100 mg kg^-1^b.wt.) | | 27.89 ± 5.2^ab^ | 258.11 ± 4.20^bc^ | 47.23 ± 1.99^c^ |
| **VII** | CCl_4_ + *Obeth* (200 mg kg^-1^b.wt.) | | 15.87 ± 3.34^b^ | 191.04 ± 6.90^d^ | 32.18 ± 3.52^cd^ |
|  | | HSD | 22.35 | 51.69 | 16.33 |
|  | | F-ratio | 3.48* | 24.55* | 46.34* |

**Values are represented as Mean ± SE (for n = 6 in each group), *level of significance p≤0.05. Means with different superscripts represent significant difference among them in all groups.**

**TABLE S11. Effect of *Obeth* on NADH cytochrome b5 and NADPH cytochrome P_450_ reductase activity among different treatment groups.**

| **Groups** | **Doses (mg kg^-1^bw)** | **NADPH cytochrome b5**  **reductase (mIU/mg/g**  **protein)** | | **NADH cytochrome P450 reductase (mIU/mg/g protein)** |
| --- | --- | --- | --- | --- |
| **I** | Control (tap water *ad libitum*) | | 86.40 ± 3.17^b^ | 37.23 ± 2.96^cd^ |
| **II** | CCl_4_(1mL/kg^-1^b.wt.) | | 188.43 ± 3.86^a^ | 73.14 ± 4.62^a^ |
| **III** | CCl_4_ + silymarin (100 mg kg^-1^b.wt.) | | 95.46 ± 3.78^b^ | 45.27 ± 1.91^bcd^ |
| **IV** | *Obeth* (200 mg kg^-1^ b.wt.) | | 79.29 ± 2.59^b^ | 41.82 ± 3.23^cd^ |
| **V** | CCl_4_ + *Obeth* (50 mg kg^-1^b.wt.) | | 161.61 ± 4.81^a^ | 58.57 ± 1.53^b^ |
| **VI** | CCl_4_ + *Obeth* (100 mg kg^-1^b.wt.) | | 153.07 ± 3.27^a^ | 50.27 ± 2.99^bc^ |
| **VII** | CCl_4_ + *Obeth* (200 mg kg^-1^b.wt.) | | 67.20 ± 2.36^b^ | 32.47 ± 4.99^d^ |
|  | HSD | 51.84 | | 14.54 |
|  | F-ratio | 16.56* | | 17.76* |

**Values are represented as Mean ± SE (for n = 6 in each group), *level of significance p≤0.05. Means with different superscripts represent significant difference among them in all groups.**

**TABLE S12. Effect of *Obeth* on CCl_4_-induced hepatic alterations in glutathione-S transferase (GST).**

| **Groups** | **Doses (mg kg^-1^bw)** | **glutathione-S-transferase (mIU/mg protein)** | |
| --- | --- | --- | --- |
| **I** | Control (tap water *ad libitum*) | | 7.6 ± 1.138^a^ |
| **II** | CCl_4_(1mL/kg^-1^b.wt.) | | 4.59 ± 0.684^b^ |
| **III** | CCl_4_ + silymarin (100 mg kg^-1^b.wt.) | | 6.93 ± 1.214^ab^ |
| **IV** | *Obeth* (200 mg kg^-1^ b.wt.) | | 7.57 ± 0.781^a^ |
| **V** | CCl_4_ + *Obeth* (50 mg kg^-1^b.wt.) | | 6.42 ± 0.85^ab^ |
| **VI** | CCl_4_ + *Obeth* (100 mg kg^-1^b.wt.) | | 6.75 ± 0.905^ab^ |
| **VII** | CCl_4_ + *Obeth* (200 mg kg^-1^b.wt.) | | 8.67 ± 1.236^a^ |
|  | HSD | 4.39 | |
|  | F-ratio | 1.62* | |

**Values are represented as Mean ± SE (for n = 6 in each group), *level of significance p≤0.05. Means with different superscripts represent significant difference among them in all groups.**

**TABLE S13. Effect of *Obeth* on CCL_4_-induced hepatic alterations in catalase (CAT), lactate dehydrogenase (LDH), and** **glutathione reductase (GR).**

| **Groups** | | **Doses (mg kg^-1^ bw)** | **CAT mIU/mg/g**  **protein** | **LDH IU/mg/g**  **Protein** | **GR IU/mg/g**  **protein** |
| --- | --- | --- | --- | --- | --- |
| **I** | Control (tap water *ad libitum*) | | 50.76 ± 1.95^ab^ | 0.077 ± 0.01^ab^ | 0.09 ± 0.01^a^ |
| **II** | CCl_4_(1mL/kg^-1^b.wt.) | | 21.92 ± 3.76^d^ | 0.03 ± 0.01^b^ | 0.03 ± 0.01^c^ |
| **III** | CCl_4_ + silymarin (100 mg kg^-1^b.wt.) | | 44.14 ± 5.11^abc^ | 0.08 ± 0.01^a^ | 0.09 ± 0.01^a^ |
| **IV** | *Obeth* (200 mg kg^-1^ b.wt.) | | 43.88 ± 2.30^bc^ | 0.07 ± 0.01^a^ | 0.08 ± 0.01^a^ |
| **V** | CCl_4_ + *Obeth* (50 mg kg^-1^b.wt.) | | 29.62 ± 3.01^cd^ | 0.05 ± 0.01^ab^ | 0.05 ± 0.01^bc^ |
| **VI** | CCl_4_ + *Obeth* (100 mg kg^-1^b.wt.) | | 40.19 ± 2.12^bc^ | 0.07 ± 0.01^ab^ | 0.07 ± 0.01^ab^ |
| **VII** | CCl_4_ + *Obeth* (200 mg kg^-1^b.wt.) | | 60.95 ± 4.11^a^ | 0.09 ± 0.01^a^ | 0.09 ± 0.01^ab^ |
|  | | HSD | 15.90 | 0.03 | 8.73 |
|  | | F-ratio | 12.45* | 4.98* | 0.03* |

**Values are represented as Mean ± SE (for n = 6 in each group), *level of significance p≤0.05. Means with different superscripts represent significant difference among them in all groups.**
